# Supplementary material for: Water Use Efficiency Spatiotemporal Change and Its Driving Analysis on the Mongolian Plateau
Source: Sensors (Basel). 2025 Apr 1;25(7):2214. doi: 10.3390/s25072214 (PMC11991396; doi:10.3390/s25072214)
Supplement: Supplementary file 1 [file sensors-25-02214-s001.zip › sensors-3427641-supplementary.pdf]

The calculation process for Theil Sen trend analysis and MK test is as follows:

$$S_{WUE} = Median\left(\frac{WUE_i - WUE_j}{j-i}\right), 1982 \le i < j \le 2018 \quad \text{(S1)}$$

If WUE is positive, it means that WUE is increasing; if it is negative, WUE is decreasing. The Mann–Kendall test was used to determine whether a trend was significant. Similarly, it considers that the sample data may not obey a certain distribution, and the method can be robust to outliers [64,65].

$$Z = \begin{cases} \frac{S+1}{\sqrt{s(S)}}, S < 0 \\ 0, \quad S = 0, \\ \frac{S-1}{\sqrt{s(S)}}, S > 0 \end{cases} \quad S = \sum_{j=1}^{n-1} \sum_{i=j+1}^{n} sgn\left(WUE_j - WUE_i\right) \quad \text{(S2)}$$

$$sgn\left(WUE_j - WUE_i\right) = \begin{cases} 1, WUE_j - WUE_i > 0 \\ 0, WUE_j - WUE_i = 0 \\ -1, WUE_j - WUE_i < 0 \end{cases}, \quad Var(S) = \frac{(2n+5)n(n-1)}{18} \quad \text{(S3)}$$

where WUE_i is the water use efficiency of year i, WUE_j is the water use efficiency of year j, sgn represents the signum function, n is the length of the time series of water use efficiency, and the value limit of statistic Z is determined as $(-\infty, +\infty)$. After determining the significance level α under the conditions of $|Z| > u_{1-\alpha/2}$, the dynamics of the trend were determined to be significant. We used $\alpha = 0.05$, and the significance of the change in the WUE time series was evaluated [66].

The mathematical process of autoregressive model (AR) is as follows:

$$Y_t = \alpha Y_{t-1} + \beta NDVIA_t + \gamma PREA_t + \delta TMEA_t + \theta SWA_t + \rho DSIA_t + \varepsilon_t \quad \text{(S4)}$$

where Yt is the anomalous WUE value in month t, namely, WUEA; εt is the residual term; α, β, γ, δ, and θ are regression coefficients of NDVIA, PREA, TEMA, SWA, and DSIA, respectively; ρ is a measure of the WUE stability of the ecosystem; and α represents the resilience of the ecosystem. The larger the α, the stronger the correlation between the current WUEA and the previous WUEA. The lower the WUE resilience of the ecosystem, the slower is the WUE recovery, and vice versa. A positive α value indicates that the current WUE anomaly's direction is aligned with that of the previous anomaly, whereas a negative value suggests a similar anomaly pattern but in the opposite direction. β, γ, δ, θ andρindicate the resistance of the WUEA to NDVIA, PREA, TEMA, SWA and DSIA in the growing season. A positive value for these parameters indicated that NDVIA, PREA, TEMA, SWA, and DSIA induced a positive WUEA response, whereas a negative value indicated that they induced a negative response. The larger the absolute value of the coefficient, the more sensitive the response of WUE to changes in environmental variables, and the weaker the resistance of WUE, and vice versa. Applying the autoregressive model to all pixels in the Mongolian Plateau, we obtained the spatial distribution of the two metrics representing the resilience and resistance of ecosystem WUE and removed the predictive variables with insignificant coefficients (p>0.05).

Based on the BFAST model results of d-h in Figure 5, we conducted a trend analysis and breakpoint detection of WUE_{Season} (from April to October) in different vegetation types on the Mongolian Plateau. The results show that the typical steppe WUE_{Season} experienced significant increase between 1982-1998 and 2008-2018, especially in eastern part of Mongolia and central part of Inner Mongolia. The broadleaf forest WUE_{Season} showed a significant increase from 1982 to 2008. For other vegetation types on the Mongolian Plateau, no significant trend shifts were observed throughout the 1982-2018 study period. This indicates that the WUE_{Season} trend changes are not obvious in meadow steppe, desert steppe, and coniferous forest vegetation types on the Mongolian Plateau. The black vertical lines represent the results of the BFAST model time series for WUE_{Season} on the Mongolian Plateau. The trends of WUE_{Season} are as follows: from 1982 to 1997, the WUE_{Season} increase trend was 0.02 per year from 1982 to 1997; from 1997 to 1998, the WUE_{Season} increase trend was 0.135 per year from 1997 to 1998; from 1998 to 2007, the WUE_{Season} increase trend was 0.002 per year from 1998 to 2007; from 2007 to 2009, the WUE_{Season} increase trend was 0.121 per year from 2007 to 2009; and from 2009 to 2018, the WUE_{Season} increase trend was 0.09 per year from 2009 to 2018.

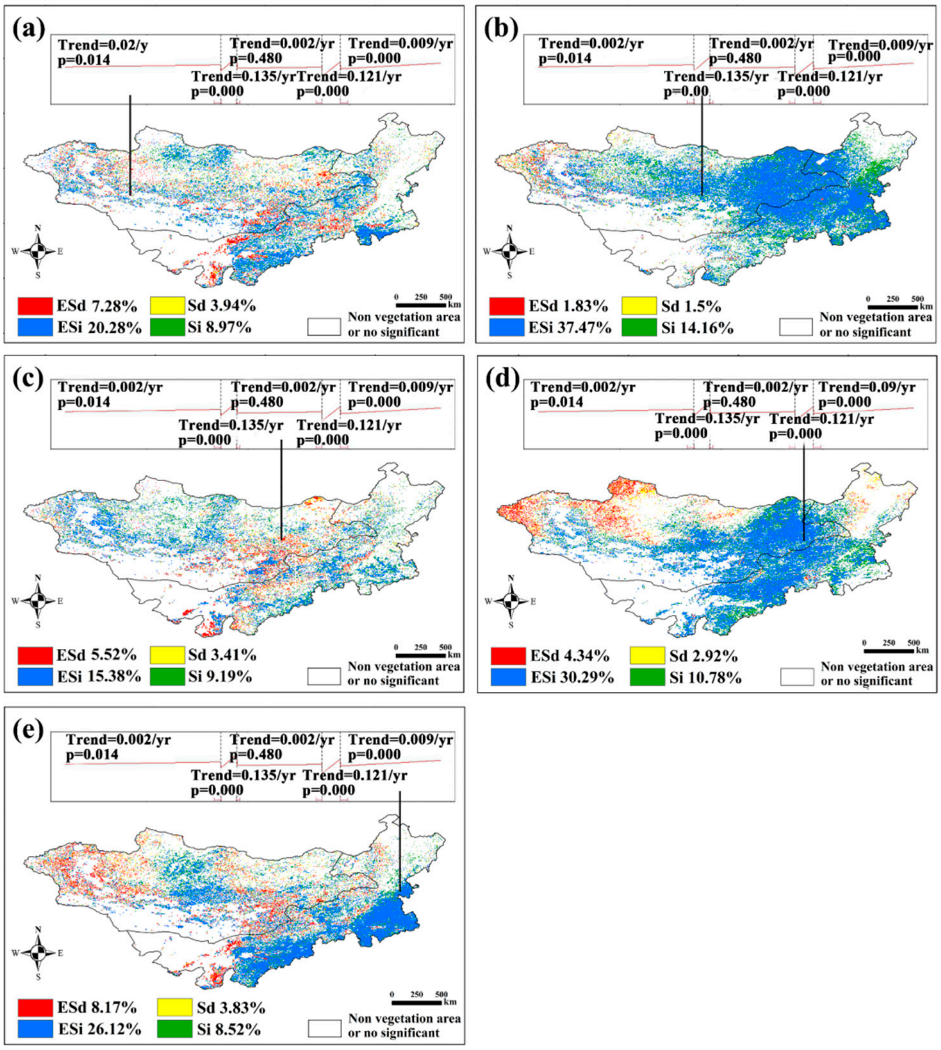

Figure S1. Spatial distribution and trend in 8-day WUESeason before and after different breakpoints: a, 1982 to 1997; b, 1997 to 1998; c, 1998 to 2007; d, 2007 to 2009; e, 2009 to 2018. Note: Sd and Si indicate a significant decrease and increase, respectively ($p<0.05$); ESd and ESi indicate an extremely significant decrease and increase, respectively ($p<0.01$).

SOS and WUE showed a positive correlation in 76.65% of the study area, with 13.4% being significant ($p<0.05$), mainly in eastern Mongolia and southern Inner Mongolia (S 2a). EOS and WUE had a positive correlation in 62.18%, with only 2.97% being significant. In general, a delayed SOS improved WUE. SOS and NPP were positively correlated in 76.46%, with 17.59% significant, mostly in central and eastern Mongolia and southern Inner Mongolia (S 2c). SOS and ET showed a positive correlation in 71.6%, with 20.72% significant ($p<0.05$), particularly in central Mongolia and central-southern Inner Mongolia (Figure 9e). EOS and NPP had a positive correlation in 56.43%, with 3.8% significant. Delaying SOS improved NPP, likely due to reduced water stress in drier springs, enhancing photosynthesis and increasing ET. The correlation between NPP, ET, and SOS was stronger than with WUE, as NPP and ET growth rates are faster, but EOS had minimal effect on WUE.

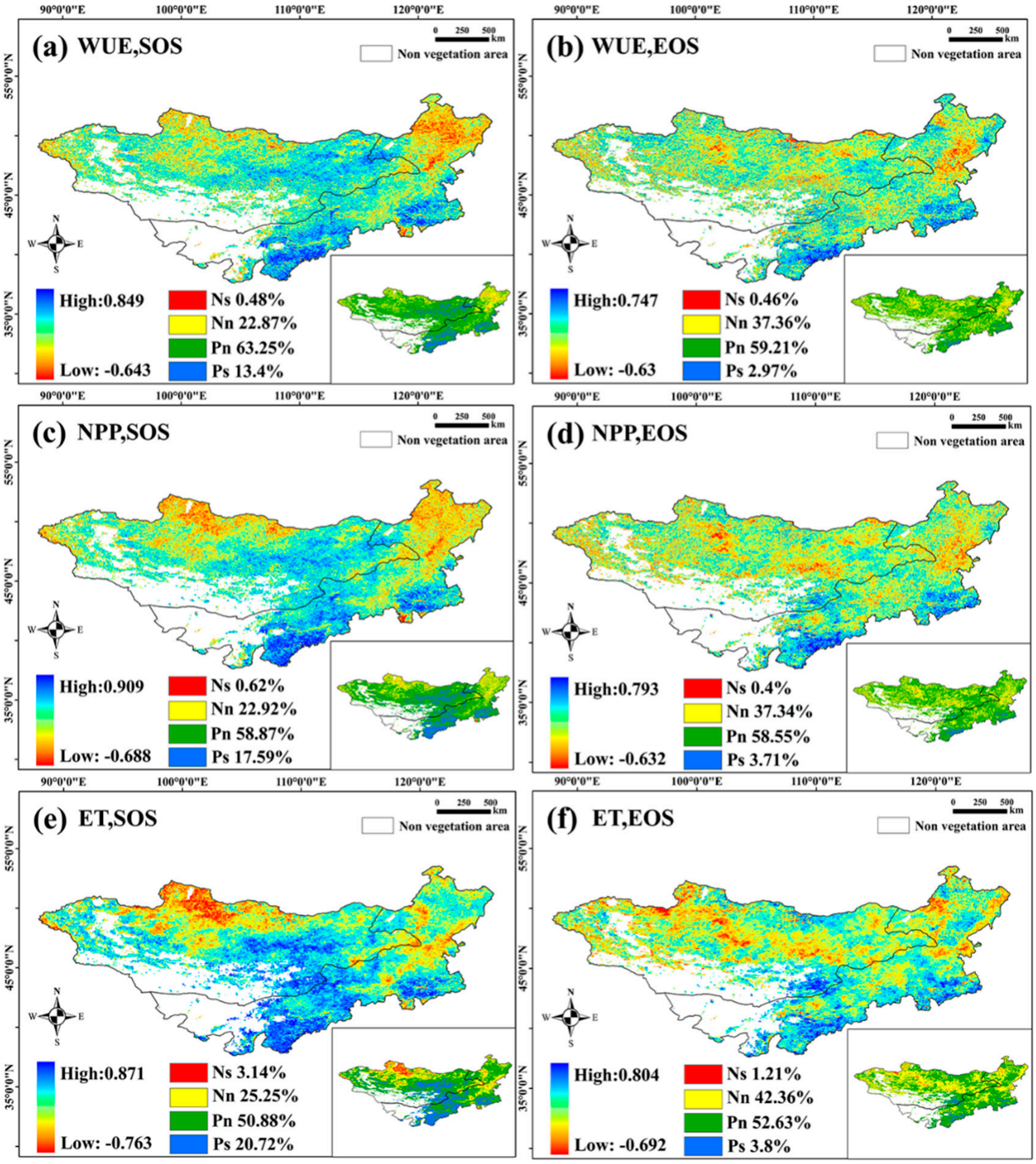

Figure S2. Partial correlation analysis between vegetation phenological parameters and WUE, NPP, and ET. a, c and e are the partial phase relationship coefficients between SOS and WUE, NPP and ET, respectively; b, d and f are the partial phase relationship coefficients between EOS and WUE, NPP and ET, respectively.
